# Changes of plasma aldosterone and angiotensin II levels in patients with ischemic cardiomyopathy combined with Type-2 diabetes mellitus and their clinical significance

**DOI:** 10.12669/pjms.38.8.5523

**Published:** 2022

**Authors:** Renmin Tang, Qian He, Min Dai, Xiulan Zou

**Affiliations:** 1 Renmin Tang, Department of Endocrinology, The People’s Hospital of China Three Gorges University, The First People’s Hospital of Yichang, Yichang 443000, Hubei, China; 2 Qian He, Department of General medicine, The People’s Hospital of China Three Gorges University, The First People’s Hospital of Yichang, Yichang 443000, Hubei, China; 3 Min Dai, Department of Endocrinology, The People’s Hospital of China Three Gorges University, The First People’s Hospital of Yichang, Yichang 443000, Hubei, China; 4 Xiulan Zou Yunji Community Healthcare Center, The People’s Hospital of China Three Gorges University, The First People’s Hospital of Yichang, Yichang 443000, Hubei, China

**Keywords:** Ischemic cardiomyopathy, Type-2 diabetes mellitus, Endocrine hormone, Left ventricular function

## Abstract

**Objective:**

To analyze the changes of plasma aldosterone (ALD) and angiotensin II (Ang II) levels in patients with ischemic cardiomyopathy (ICM) combined with Type-2 diabetes mellitus (T2DM) and their clinical significance.

**Methods:**

Sixty-eight patients with ICM combined with T2DM and fifty-two patients with simple ICM treated in the People’s Hospital of Three Gorges University/the First People’s Hospital of Yichang from February 2018 to February 2021 were selected as observation group and control group, respectively. All the patients had intervention with the same neuroendocrine hormone regime. The plasma ALD and Ang II and left ventricular function indexes (left ventricular ejection fraction (LVEF), left ventricular end-diastolic diameter (LVEDD) and left ventricular end-systolic diameter (LVESD)] were measured and compared between the two groups before and after treatment. The correlations of plasma ALD and Ang-II with left ventricular function before treatment were analyzed using Pearson’s correlation analysis. The diagnostic efficacy of plasma ALD and Ang-II in ICM and T2DM was evaluated by the receiver operating characteristic (ROC) curve.

**Results:**

Before treatment, the plasma ALD and Ang II levels in the observation group were (184.42 ± 56.75) ng/L and (46.68 ± 12.16) ng/L, respectively, which were significantly higher than those in the control group [(165.03 ± 45.67) ng/L and (39.70 ± 10.69) ng/L, p< 0.05]. Compared with before treatment, ALD level increased significantly in the observation group while decreased significantly in the control group after treatment (*p<* 0.05). After treatment, Ang-II level reduced significantly while LVEF increased significantly in both groups (*p<* 0.05). After treatment, plasma ALD and LVESD in the observation group were significantly higher than those in the control group (*p<* 0.05), but plasma Ang II level, LVEF and LVEDD showed no statistically significant differences between the two groups (*p>* 0.05). Before treatment, plasma ALD and Ang II were negatively correlated with LVEF (*p<* 0.05). Before treatment, the area under the curve (AUC) of plasma ALD and Ang II levels in diagnosing ICM combined with T2DM were 0.689 and 0.704, respectively.

**Conclusion:**

The plasma levels of ALD and Ang-II in patients with ICM combined with T2DM increase significantly, and their diagnostic value is not high. Compared with patients with simple ICM, the decrease in plasma ALD and Ang- II levels is less obvious after the same intervention, but it is still conducive to the improvement of cardiac function.

## INTRODUCTION

Ischemic cardiomyopathy (ICM) is typically characterized by myocardial ischemia, coronary atherosclerosis and coronary microvascular lesions as its pathogeny. Especially, coronary atherosclerosis, known as late-stage coronary heart disease (CHD), is prone to cause heart failure (referred to as HF) and even sudden death.^1^-^4^ Type-2 diabetes mellitus (T2DM) is an independent risk factor for CHD.^5^ In recent years, the incidence of ICM has increased with the increase in CHD. Clinically, ICM combined with T2DM is common, and its risk of HF is significantly higher compared with patients with simple ICM.^6^,^7^ However, the current reports on endocrine hormones in ICM combined with T2DM are few. Therefore, in this study, the changes in endocrine hormones-aldosterone (ALD) and angiotensin II (Ang-II) in patients with ICM combined with T2DM and their correlations with left ventricular function were mainly analyzed, so as to guide the clinical treatment of ICM combined with T2DM.

## METHODS

Sixty-eight patients with ICM combined with T2DM treated in the People’s Hospital of Three Gorges University/the First People’s Hospital of Yichang from February 2018 to February 2021 were selected as observation group.

### Ethical Approval:

The study was approved by the Institutional Ethics Committee of The People’s Hospital of China Three Gorges University/the First People’s Hospital of Yichang on June 18^th^,2021(No:2021035), and written informed consent was obtained from all participants.

### Inclusion criteria:

Patients diagnosed as ICM by echocardiography and coronary angiography; patients presenting angina pectoris and cardiac enlargement; patients with T2DM diagnose meeting the relevant criteria^8^ patients with good cooperation; patients with complete related data; patients signing the informed consent.

### Exclusion criteria:

Hypertension, primary cardiomyopathy and other diseases leading to cardiac enlargement and HF; liver and kidney dysfunction; cardiogenic shock; severe pulmonary diseases; arrhythmia caused by related drugs; malignant tumors; incomplete data. During the same period, fifty-two patients with simple ICM were included as control group. They were diagnosed by relevant examinations and had normal blood glucose, and the exclusion criteria were the same as above. No statistically significant differences were found in gender, age, body mass index (BMI), hypertension, hyperlipidemia or smoking history between the two groups (*p>* 0.05).Table-I.

**Table-I:** Comparison of general data between the two groups.

Group	N	Male/female	Age (years)	BMI (kg/m^2^)	Hypertension	Hyperlipidemia	Smoking history
Observation group	68	40/28	65.14 ± 6.50	24.25 ± 2.38	23	29	35
Control group	52	28/24	63.28 ± 7.85	23.93 ± 3.05	13	16	25
χ^2^ or t		0.337	1.419	0.646	0.097	0.222	0.679
p		0.561	0.158	0.520	0.755	0.637	0.410

### Treatment methods:

All the patients received routine drug intervention for ICM such as diuretics and aldosterone antagonists, and were given symptomatic treatment such as lowering blood glucose according to the patients’ conditions. In addition, both groups were treated with the same neuroendocrine hormone regime: β-receptor blockers (such as betaloc) + angiotensin-converting enzyme inhibitors (such as fosinopril or captopril). The initial dose was determined according to the patients’ conditions, and it should be increased gradually and tolerable the patients. Betaloc (100 mg/d) was used in 20 patients in the observation group and 18 patients in the control group, fosinopril in 30 patients in the observation group and 25 patients in the control group, and captopril in 38 patients in the observation group and 27 patients in the control group. The intervention was maintained for 6 months.

### Detection Indexes:

After admission (before treatment) and months after treatment, fasting peripheral blood was collected at 7 a.m. on the next day. The plasma for ALD detection was anticoagulated with heparin, and that for Ang-II was anticoagulated with ethylenediaminetetraacetic acid. They were both measured with radioimmunoassay using the kit purchased from Beijing North Institute of Biotechnology Co., Ltd. according to the instructions.

### Echocardiography:

After admission (before treatment) and six months after treatment, the left ventricular function indexes were measured using HD1500 color Doppler echocardiography, including left ventricular ejection fraction (LVEF), left ventricular end-diastolic diameter (LVEDD) and left ventricular end-systolic diameter (LVESD).

### Statistical Analysis:

The date was processed using SPSS 20.0. The enumeration data were expressed as % and analyzed by the *χ^2^* test. The measurement data were expressed as x̄±s and analyzed with the *t* test. The efficacy of plasma ALD and Ang-II levels in the diagnosis of ICM combined with T2DM was analyzed using the ROC curve. The correlation was analyzed by the Pearson’s correlation analysis. α = 0.05 was considered as statistically significant.

## RESULTS

The plasma ALD level in the observation group was significantly higher than that in the control group before and after treatment, and plasma Ang II level in the observation group was significantly higher than that in the control group before treatment (*p<* 0.05). After treatment, no statistically significant difference was found in plasma Ang II level between the two groups (*p>* 0.05).Table-II.

**Table-II:** Comparison of plasma ALD and Ang II levels between the two groups before and after treatment (x̄ , ng/L)

Group	N	ALD		Ang II	
	
		Before treatment	After treatment	Before treatment	After treatment
Observation group	68	184.42 ± 56.75	228.25 ± 87.69*	46.68 ± 12.16	32.20 ± 8.76*
Control group	52	165.03 ± 45.67	134.37 ± 62.16*	39.70 ± 10.69	34.41 ± 10.05*
*t*		2.014	6.559	3.281	1.285
*p*		0.046	< 0.001	0.001	0.201

***Notes:*** Compared with before treatment in the same group,

* p < 0.05.

**Fig.1:**
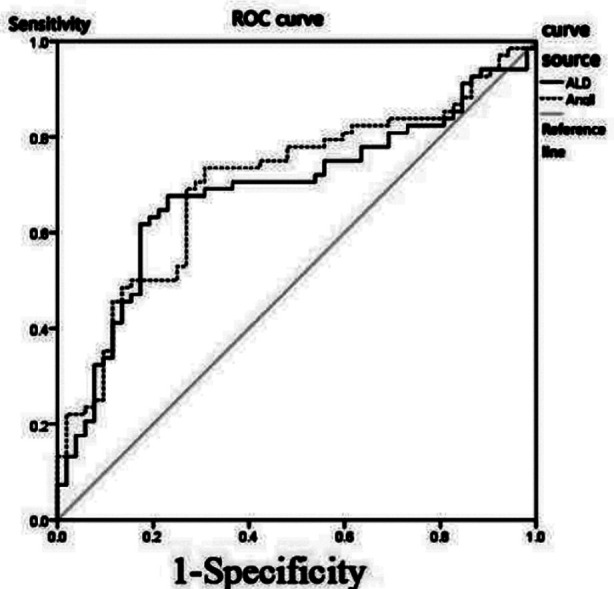
ROC curve of plasma ALD and Ang II levels in diagnosing ICM combined with T2DM before treatment.

Diagnostic value of plasma ALD and Ang II levels in ICM combined with T2DM before treatment: According to the ROC analysis, the area under the curve (AUC) of plasma ALD in diagnosing ICM combined with T2DM was 0.689 before treatment. When the cutoff value was 199.80 ng/L, the sensitivity and specificity were 67.65% and 76.92%, respectively. Table-III, Fig.1

**Table-III:** Diagnostic efficacy of ALD and Ang II levels in ICM combined with T2DM before treatment.

Index	AUC	Cutoff value	Youden index	Sensitivity	Specificity
ALD (ng/L)	0.689	199.80	0.45	67.65	76.92
Ang II (ng/L)	0.704	42.77	0.43	73.53	69.23

Before treatment, LVEF, LVEDD and LVESD showed no statistically significant differences between the two groups (*p>* 0.05). After treatment, LVEF in both groups increased significantly than that before treatment (*p<* 0.05). After treatment, LVESD in the control group reduced significantly compared with that before treatment, and was significantly lower than that in the observation group (*p<* 0.05).Table-IV.Pearson’s correlation analysis revealed that before treatment, plasma ALD and Ang II levels were negatively correlated with LVEF (*p<* 0.05), but not correlated with LVEDD or LVESD (*p>* 0.05).Table-V.

**Table-IV:** Changes of left ventricular function indexes in the two groups before and after treatment (x̄±s )

Groups	N	LVEF (%)		LVEDD (mm)		LVESD (mm)	

		Before treatment	After treatment	Before treatment	After treatment	Before treatment	After treatment
Observation group	68	51.10 ± 8.36	54.36 ± 8.65*	51.23 ± 4.76	50.96 ± 5.13	36.76 ± 5.38	35.52 ± 4.80
Control group	52	51.45 ± 8.49	57.30 ± 8.13*	51.69 ± 5.72	50.41 ± 5.58	36.50 ± 5.46	32.48 ± 4.65*
t		0.226	1.893	0.481	0.560	0.261	3.485
p		0.822	0.061	0.632	0.576	0.795	0.001

***Notes:*** Compared with before treatment in the same group,

* p< 0.05.

**Table-V:** Correlations of plasma ALD and Ang II levels with left ventricular function (r).

Index	LVEF	LVEDD	LVESD
ALD	-0.560*	0.125	0.212
Ang II	-0.653*	0.146	0.228

* ***Notes:*** p< 0.05.

## DISCUSSION

The cardiac autonomic nervous function in ICM combined with T2DM is abnormal, characterized by reduced vagal tone and increased sympathetic tone. During HF progression, the sympathetic nerve and vagus nerve are damaged in varying degrees, especially the vagus nerve. With imbalance, it will lead to malignant arrhythmia, aggravate HF, decrease heart rate variability, increase the risk of ventricular arrhythmia, and even cause sudden death.^9^-^12^

The onset of ICM is closely related to atherosclerosis, and the occurrence and development of atherosclerosis and thrombosis are related to inflammation.^13^,^14^ Inflammation and myocardial ischemia can both lead to abnormal hemodynamics and activate renin angiotensin system (RAS) and neuroendocrine system^15^,^16^, which can promote cardiac remodeling and relieve HF. However, if the time of neuroendocrine reaction is too long, the condition may be aggravated.^17^ ALD and Ang II are a part of RAS. In addition to distribution in the circulatory system, RAS is also distributed in the heart, kidney and other tissues. Under normal condition, this system has multiple functions, such as maintaining electrolyte and body fluid balance, regulating blood pressure and maintaining normal development of the cardiovascular system.^18^,^19^ RAS can not only lead to local coronary artery contraction, but also enhance myocardial contractility by improving calcium channel permeability and calcium influx.^20^ Additionally, during myocardial ischemia, RAS can promote DNA synthesis and increase fibroblasts, which is prone to cause ventricular fibrosis, affect myocardial metabolism, aggravate ischemic myocardial damage and increase the incidence of ventricular arrhythmia.^21^ After the activation of RAS, the plasma levels of ALD and Ang-II increase. Among them, Ang is produced by renin acting on angiotensinogen, and then stimulates the glomerular zone of the adrenal cortex to promote the production and secretion of ALD. High blood glucose can increase the expression of Ang-II in the proximal renal tubules, and abnormal products of glucose metabolism can also increase RAS-related components in the kidney. The mechanism may include^22^,^23^: high blood glucose can produce Ang II through the non-angiotensin converting enzyme pathway; and high blood glucose can promote the production of advanced glycation end-products in oxidative stress and produce Ang-II through chymase. In this study, the results showed that the plasma levels of ALD and Ang-II in patients with ICM combined with T2DM increased significantly before treatment. This is caused by that both ICM and T2DM have effects on RAS, further activating this system, so that the plasma levels of ALD and Ang II increase more significantly. Additionally, ROC analysis revealed that the diagnostic efficacy of plasma ALD and Ang II levels in ICM combined with T2DM is not high, with the AUC of about 0.7 and relatively low value. It is suggested that RAS may be involved in the pathogenesis of ICM combined with T2DM. However, the main pathogenesis of ICM and T2DM is not RAS, which is activated by hemodynamic abnormalities such as inflammation and myocardial ischemia, and is an indirect effect. Therefore, it is necessary to evaluate ICM and T2DM in combination with other examinations in clinic.

In addition, our study found that after treatment, the plasma ALD level of patients with ICM combined with T2DM increased further, while that of the patients without T2DM decreased significantly, which may be related to the escape of ALD and the increase in Ang-converting enzyme activity in patients combined with T2DM. It can be seen that patients with ICM combined with T2DM need to be treated with ALD antagonists to inhibit the proliferation of collagen fibers caused by ALD, which is conducive to blocking cardiac fibrosis. Moreover, it was found that after treatment, the plasma Ang-II level decreased significantly, which resulted from the increase of Ang-converting enzyme activity in patients with T2DM. The treatment with Ang-converting enzyme inhibitors could effectively reduce the level of Ang-II, which is conducive to the improvement of cardiac function. Furthermore, LVEF after treatment increased significantly in the two groups compared with that before treatment, and no significant difference was found between the two groups, which may be related to the application of Ang-converting enzyme inhibitors. It was also found that the LVESD of patients with ICM combined with T2DM was significantly larger than that of patients with simple ICM after treatment, which may be related to the RAS contracting local coronary artery and affecting cardiac contractility. Finally, plasma ALD and Ang II were found to be negatively correlated with LVEF, but not correlated with LVEDD or LVESD, which may be relevant to the correlation between HF and LVEF.

### Limitations of the study:

Due to the lack of research on the correlations of ALD and Ang II with left ventricular function, and the small sample size of this study, the correlations need to be further analyzed in the future.

## CONCLUSION

ICM combined with T2DM activates RAS, and significantly increase plasma ALD and Ang-II levels. However, the value of plasma ALD and Ang-II in the diagnosis of ICM combined with T2DM is not high, and the clinical diagnosis should be based on other examinations and symptoms. Additionally, plasma ALD and Ang II are correlated with LVEF. Although the same neuroendocrine hormone regimen is less effective than simple ICM, it is still conducive to improving the cardiac function of patients.

### Authors’ Contributions:

**RT &** **XZ:** Designed this study, prepared this manuscript, are responsible and accountable for the accuracy and r integrity of the work.

**QH:** Collected and analyzed clinical data.

**MD:** Data analysis, significantly revised this manuscript.
